# Naming of Parts

**DOI:** 10.1177/21501351261418653

**Published:** 2026-03-09

**Authors:** Niraj N. Pandey, Robert H. Anderson

**Affiliations:** 1 Cardiothoracic Sciences Center, 28730 All India Institute of Medical Sciences, New Delhi, India; 2 Institute of Biomedical Sciences, 5994 Newcastle University, Newcastle-upon-Tyne, UK

**Figure 1.**
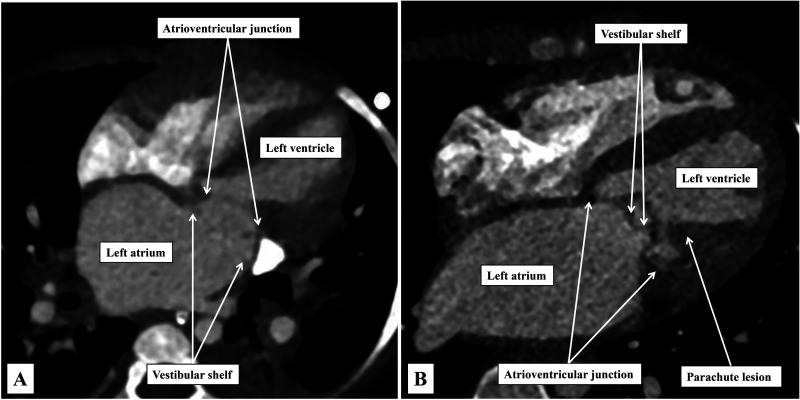
The images, taken from computer tomographic angiographic datasets, show the differences between the vestibular (panel A) and the intramitral (panel B) variants of so-called “supramitral” valvar obstruction. Note the presence of the parachute malformation of the papillary muscles of the mitral valve in panel B.

“Naming of parts” was a poem written in World War II by Henry Reed.^1^ It describes the account given by the instructor for the disassembling of a rifle, detailing all the parts, including those that the recruits do not have, since the manual from which they are working is out of date. The recruits would have understood what they were required to do because the names given to the different parts were specific, including the parts that they did not have. It is unfortunate that such specificity, as yet, does not relate to the naming of the components of the heart, particularly when the heart in question is malformed. But we are moving in the appropriate direction. In this issue of the Journal,^2^ part of the surgical team working at the All India Institute of Medical Sciences in New Delhi (AIIMS) has analyzed their experience treating the lesions initially described as representing “congenital disease of the left side of the heart.”^3^ For us, from the stance of phenotypic diagnosis, the most important message from their analysis is the need to distinguish between the vestibular and intravalvar variants (Figure 1). For quite some time, the lesions were simply interpreted as representing “supravalvar mitral obstruction.” In a relatively large surgical series, for example, the authors, in keeping with previous experience, had suggested that the lesion should be distinguished from subdivision of the left atrium.^4^ Although citing the investigation of another relatively large series reported from Rome,^5^ in which the true “supramitral” vestibular lesions had already been recognized as different from the intravalvar variants, the group from Indiana made no mention of this fact.^4^ From the stance of anatomy, it is very much the case that the vestibular variant is another example of subdivision of the left atrium (Figure 1, panel A), rather than a problem involving the mitral valve (Figure 1, panel B). As we assess the earliest descriptions, nonetheless, it is easy to understand why, until recently, difficulties remained in distinguishing between the two phenotypes.

The lesion was first encountered in the autopsy examination of an infant who died at the age of 15 months. The account provided is remarkably precise.^3^ In addition to the expected findings of mitral stenosis, the author found “a perforated diaphragm, resembling the diaphragm of the microscope, formed by a circular membrane, the base of which was attached to the bases of the mitral flaps.” This is clearly a description of the intravalvar variant (Figure 1, panel B). The subsequent description of a comparable entity, over half a century later, provides an understanding of from where the problems of precise diagnosis began to arise.^6^ The obstructing lesion was again described as a “ring of fibrous tissue.” It was said to be positioned “just above the base of the valve,” but “protruded for 3 mm into the left atrial cavity.” Although the authors considered the ring to be “above the mitral valve,” the histological section they provided shows the lesion arising from the leaflet of the mitral valve, albeit close to the atrioventricular junction. As such, it is located within the vestibule of the left atrium, rather than being part of the atrioventricular junction itself. In terms of the anatomy described, the account is directly comparable to that provided by Fisher.^3^ It is arguably the study conducted echocardiographically by Sullivan and colleagues that first emphasized the presence of the two phenotypic variants now recognized by the surgeons from AIIMS.^7^ Subsequent to the echocardiographic recognition,^7^ the team of Carpentier, working at Hôpital Broussais in Paris, emphasized that, although usually described as being supravalvar, the obstructing lesion was often within the funnel of leaflet tissue of the mitral valve.^8^ The group from Children's Memorial Hospital in Chicago, when describing their own experience, cited the definition as offered by the Parisians, and recognized one of their patients as exhibiting a “midvalvar” lesion.^9^ The difference between the intravalvar and vestibular variants was then endorsed by Toscana and colleagues.^5^

As is now described by the group working in New Delhi, it is possible for the surgeon to identify the lesion itself, be it intravalvar or vestibular, and remove it, leaving behind the native mitral valve. This was achieved successfully in all but one of the 51 patients making up the Indian cohort, with the unsuccessful patient dying due to ventilator-associated sepsis. An additional three patients died during the period of follow-up. Brown and colleagues had achieved immediate success in all of their 27 patients,^4^ but they lost five during subsequent follow-up. The group from Rome had operated on 13 patients,^5^ and all of their patients remained alive. The various reports indicate that the location of the obstructive lesion has a significant relationship to the arrangement of the mitral valve itself. This is because, when the lesion is intravalvar, it is likely to be accompanied by one of more of the lesions that make up the so-called Shone complex.^10^ A so-called “supravalvular” lesion was found in all 8 of the hearts initially described by Shone and his colleagues.^10^ The lesion itself, however, was described as “a circumferential ridge of connective tissue that arises at the base of the atrial surfaces of the mitral leaflets and protrudes into the inlet of the mitral valve.” To our eyes, this is an excellent description of the intravalvar variant. It is not surprising that it should be found in association with abnormal papillary muscles, a subaortic shelf, and coarctation, although rarely all at the same time. The vestibular variant, in contrast, although less directly related to the mitral valve, can be found, as exemplified by the experience from AIIMS, in the setting of multiple associated anomalies.

Throughout the descriptions of the obstructive lesion, be it vestibular or intravalvar, it is frequent to find it described as being “membranous.” In this regard, the group from Rome had commented on the difference in the structure of the obstructive lesion itself depending on its location. The vestibular variant was described as a “fibrous, shelf-like membrane,” whereas the intravalvar variant was considered to be a “thin membrane within the mitral tunnel.” But both were considered to be “membranous,” as is usually the shelf producing discrete subaortic obstruction. The group from AIIMS was also comfortable when describing the lesions as being “membranous.” It follows that, for full precision, it is necessary to provide details of the quality of the “membrane.” Of note is that, in the series reported from AIIMS,^2^ a significant majority had vestibular membranes, as was the case in the small series reported from Chicago.^9^ In the series reported by Sullivan and colleagues,^7^ in contrast, and that of Toscana and associates,^5^ it was the intravalvar variant that was more frequent. In the studies reported to date, diagnosis and differentiation has depended in the greater part on echocardiography. Ongoing experience at AIIMS now points to the added value of interrogation using computed tomographic angiography (Figure 1). The technique has the added value of showing the details of any and all associated intracardiac anomalies.
